# Supplementary material for: The ontogenetic transformation of the mesosaurid tarsus: a contribution to the origin of the primitive amniotic astragalus
Source: PeerJ. 2016 May 17;4:e2036. doi: 10.7717/peerj.2036 (PMC4878385; doi:10.7717/peerj.2036)

The study of a large number of mesosaur specimens from Uruguay and Brazil has provided interesting information about the changes that take places during the ontogeny of the group. One of the most amazing discovering was an almost complete, very small and beautiful preserved specimen, which was interpreted as a non-hatched Mesosaurus (Piñeiro et al. 2012), probably close to complete its development, although some indications of its foetal stage are clear: the porous and poorly ossified nature of the bones, the structure of the teeth, including uncomplete and bifurcated elements, the incipient development of the front limbs, particularly the hands, contrasting with the high size of the feet, which preserve what appears to be an astragalus, as the only tarsal element observed. This specimen consists therefore, the only evidence of reproduction in an early amniote and allows us to reconstruct the earliest stages of one of the most complete ontogenetic transition known for a basal component of the group. The purported sutured astragalus is formed by three visible bones (intermedium, tibiale and c3), but indeed it may have been an earlier fusion of the tibiale to c4 as shown by the condition in ancestral tetrapods (Holmes, 1984; Carroll, 1970). Below, we present additional photographs of the non-hatched specimen and particularly, of the area of feet location that show the composite structure of the possible foetal astragalus, which could be the strongest evidence in support to the traditional Peabody's (1951) hypothesis about the origin of the amniotic tarsus.

References.

Carroll RL. 1970. The ancestry of reptiles. Philosophical Trans-actions of the Royal Society of London B 257:267–308.

Holmes R. 1984. The Carboniferous amphibian Proterogyrinus scheelei Romer, and the early evolution of tetrapods. Philosophical Transactions of the Royal Society of London, B 306:431–524.

Peabody FE. 1951. The origin of the astragalus of reptiles. Evolution 5: 339–344.

Piñeiro G, Ferigolo J, Meneghel M & Laurin M. 2012b. The oldest known amniotic embryos suggest viviparity in mesosaurs. Historical Biology 24 (6):630–640.

Supplemental Figure 1

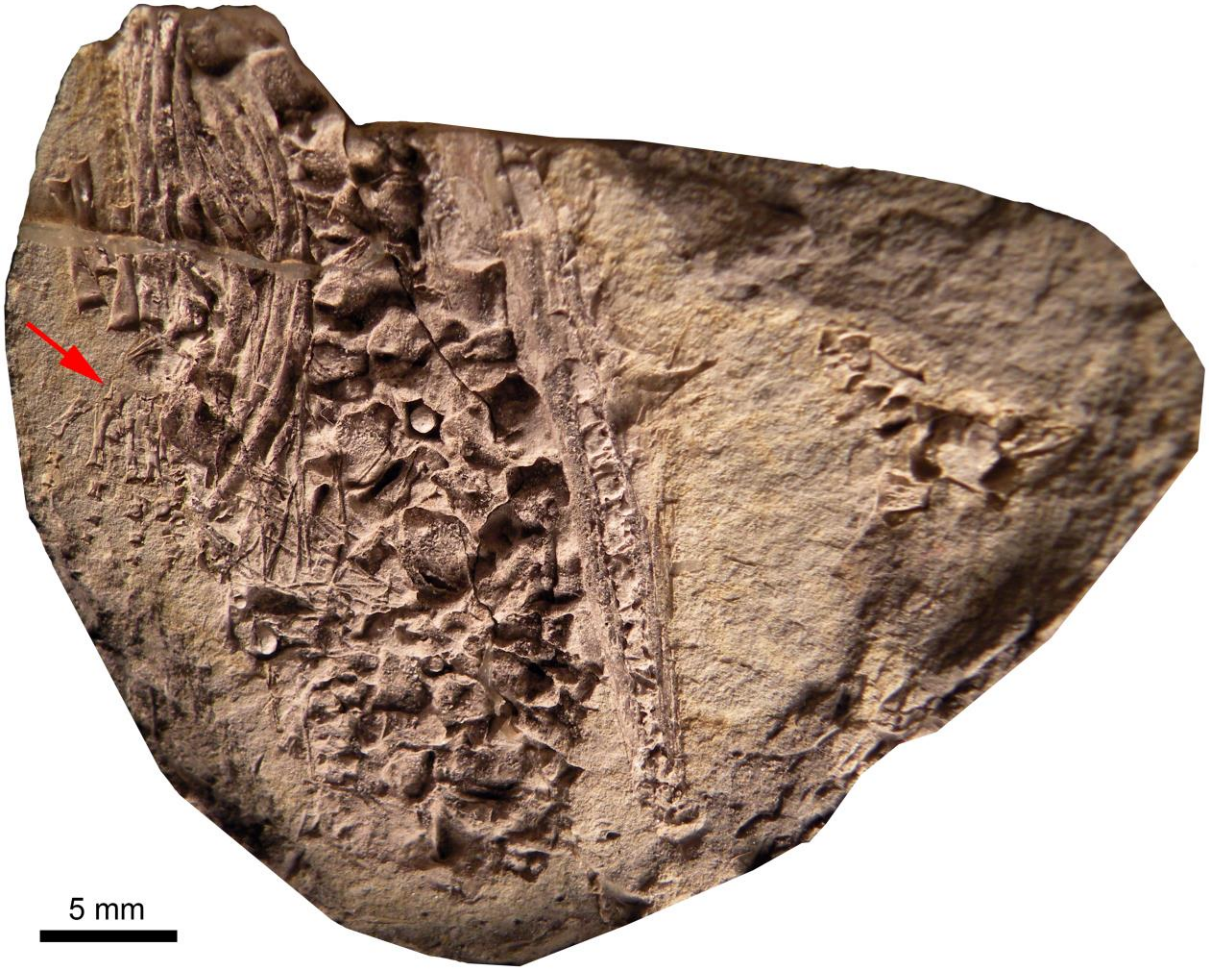

Supplemental Figure 2

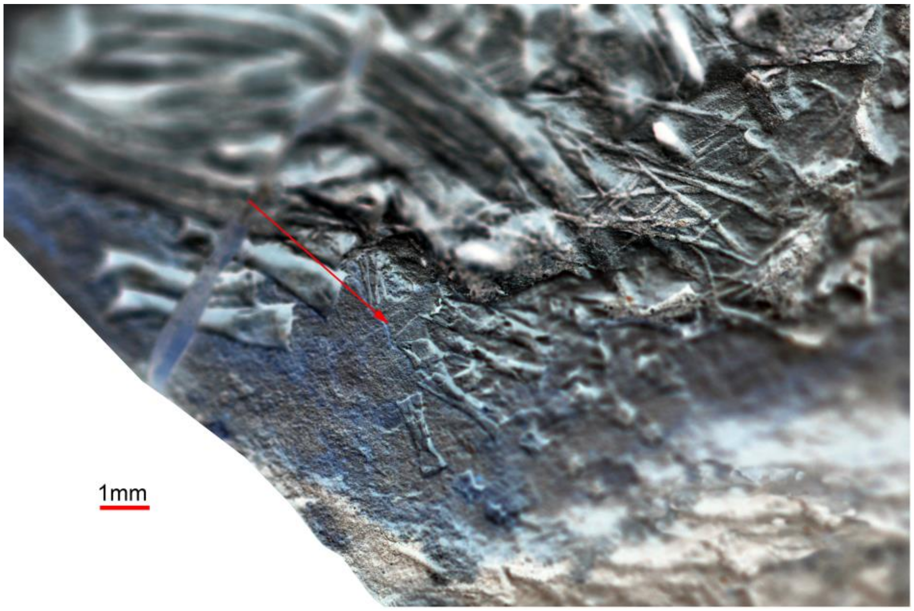

Supplemental Figure 3

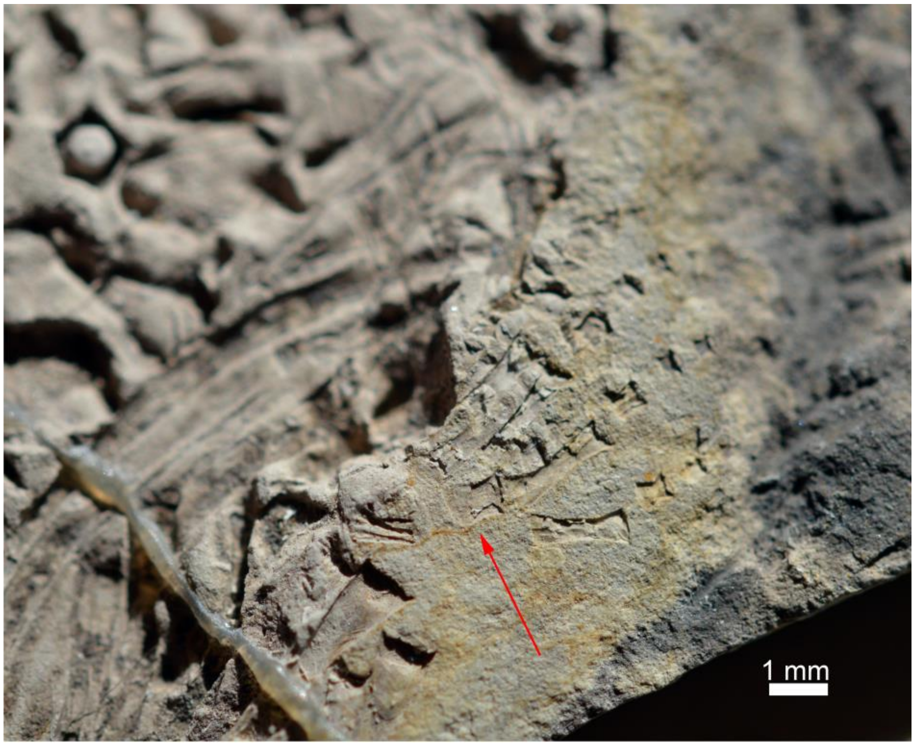

Supplement: Supplemental Information 1 — Figures of non-hatched Mesosaurus tenuidens. Close view of the feet area. Arrows indicates the position of the sutured astragalus. [file peerj-04-2036-s001.pdf]
